# First spin-resolved electron distributions in crystals from combined polarized neutron and X-ray diffraction experiments

**DOI:** 10.1107/S2052252514007283

**Published:** 2014-04-14

**Authors:** Maxime Deutsch, Béatrice Gillon, Nicolas Claiser, Jean-Michel Gillet, Claude Lecomte, Mohamed Souhassou

**Affiliations:** a CRM2, CNRS-Université de Lorraine, BP 70239, Vandoeuvre-lès-Nancy CEDEX, 54506, France; b LLB, CEA-CNRS, CEA Saclay, Gif-sur-Yvette, 78180, France; c SPMS, CNRS-Ecole Centrale des Arts et Manufactures, Grande Voie des Vignes, Châtenay-Malabry CEDEX, 92295, France

**Keywords:** multipole refinement, charge and spin densities, joint refinement, molecular magnetic materials, magnetization density, polarized neutron diffraction

## Abstract

Since the 1980s it has been possible to probe crystallized matter, thanks to X-ray or neutron scattering techniques, to obtain an accurate charge density or spin distribution at the atomic scale. Despite the description of the same physical quantity (electron density) and tremendous development of sources, detectors, data treatment software *etc.*, these different techniques evolved separately with one model per experiment. However, a breakthrough was recently made by the development of a common model in order to combine information coming from all these different experiments. Here we report the first experimental determination of spin-resolved electron density obtained by a combined treatment of X-ray, neutron and polarized neutron diffraction data. These experimental spin up and spin down densities compare very well with density functional theory (DFT) calculations and also confirm a theoretical prediction made in 1985 which claims that majority spin electrons should have a more contracted distribution around the nucleus than minority spin electrons. Topological analysis of the resulting experimental spin-resolved electron density is also briefly discussed.

## Abstract 2

A method to map spin-resolved electron distribution from combined polarized neutron and X-ray diffraction is described and applied for the first time to a molecular magnet and it is shown that spin up density is 5% more contracted than spin down density.

## 1. Introduction   

We learn from quantum physics that, in a crystal or a molecule, electrons are smoothly distributed according to a probability distribution law. In a bonded system, this electron distribution is significantly different from that of the mere sum of non-interacting atoms (the ‘independent atoms model’, IAM). This difference is indeed the key quantity to understand (and predict) not only chemical bonding mechanisms but also the resulting properties of molecules and crystals. When the system under study possesses magnetic properties, the charge distribution alone is not sufficient. One needs then to gain better knowledge of how the electrons, depending on their spin state, spread around and between the nuclei.

The purpose of this article is to demonstrate that the experimental determination of the electron position probability density has now reached a point where it has become possible to model and separate up and down spin carrier contributions to the distribution. However, this type of result is only possible by combining different types of experiments and interpreting them through a common model (Deutsch *et al.*, 2012 ▶). In this paper we report on the first successful experimental reconstruction of a spin-resolved electron density distribution and some insights obtained in this way for a model molecular magnetic crystal.

## 2. X-rays for an electron probability density distribution   

Since the pioneering experiments conducted by Laue a hundred years ago it has been established that an X-ray diffraction (XRD) experiment provides a measurement of the Fourier coefficients (named ‘structure factors’, 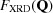) of the electron distribution in the crystal. While the Fourier decomposition is infinite, the accuracy of an electron density reconstruction is limited by the experimental resolution in reciprocal space, typically around 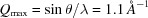.

Owing to such resolution limitations and the need for experimental corrections, the electron distribution is often reconstructed by means of a model. The most popular one is the ‘deformed pseudo-atoms’ model proposed by Hansen & Coppens (1978 ▶). This model assumes that the electron probability density distribution, denoted hereafter as 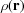, can be fairly expressed as a sum of densities centred on each nucleus – hence the name ‘pseudo-atoms’. Initially, each of these atomic contributions is identical to what it would be if the atoms were isolated. In particular, the atomic electron density is assumed to be locally isotropic and neutral. This is indeed a common hypothesis that is always used for crystal structure determination. However, upon chemical bond formation and subsequent charge transfers, the outer part of these pseudo-atomic electron densities will need to expand (or contract) and distort in some specific directions. For each of these pseudo-atoms, an expansion over a limited set of real spherical harmonics functions 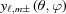 has been found to efficiently account for the amount of information provided by X-ray diffraction experiments. Therefore, the global contribution of a given atom *i* to the density distribution within the Hansen–Coppens (H–C) model is
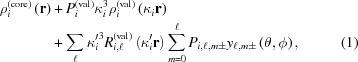
where 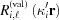 are radial Slater-type functions.

The best suited values for extension/contraction 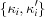 and valence and multipole population 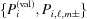 parameters are determined to match experimental data 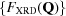.

This particular model for charge density has encountered such tremendous success since the 1980s that high-resolution XRD is now frequently employed to analyze chemical bonds and more generally the charge redistribution due to molecule or crystal formation. This experimental approach to electron probability distribution has become extensively used and was developed to calibrate advanced quantum chemistry computations. Such a landmark is of course essential, especially if the aim is to extend the theoretical modelling to a set of systems which cannot yet be studied experimentally (Gatti & Macchi, 2012 ▶).

## 3. Polarized neutrons for unpaired electron probability distribution   

As a consequence of this success, the quest for a deeper understanding of mechanisms responsible for spin magnetism has led to the use of the same approach to reconstruct electron magnetization distribution in crystals from polarized neutron diffraction (PND) data.

While XRD and non-polarized neutron diffraction data consist of integrated intensities of Bragg reflections, PND measures ‘flipping ratios’ (hereafter denoted 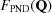). They are defined as the ratio between the diffracted intensities for spin up and spin down incident neutrons. This technique thus aims to identify regions of the crystal unit cell where an electron spin majority exists and how it is distributed. PND gives access to the magnetization density that is the sum of the pure spin density and the orbital contribution (Schweizer, 2006 ▶).

In a PND data collection, the explored reciprocal space domain is generally more limited than its XRD counterpart because of the restricted access to neutron beam time and the available range of experimental geometries (the sample is submitted to a strong magnetic field, typically 5 T). Thus, PND is often limited to the domain of 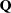 for which a significant magnetic contribution is expected, *i.e.* *Q*_max_ is 0.5 Å^−1^ for 3*d* transition metals. Moreover, as opposed to structural data collections which concern all the existing reflections in a given domain of 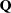, PND measurements are performed only for the strong nuclear reflections for which good experimental accuracies may be achieved for the flipping ratios. Therefore, only ∼ 10% of all reflections in a given reciprocal domain are measured by PND.

Despite experimental difficulties that hinder its resolution, PND has provided a wealth of information to the magnetism community. In fact, the spin density visualization provides direct information about the magnetic interaction pathways and the sign of the spin density distribution along this pathway, *i.e.* a crucial test of the nature of the mechanism (Pontillon *et al.*, 1999 ▶; Pillet *et al.*, 2001 ▶).

## 4. The combination challenge in determining a spin-resolved electron density distribution   

From the above considerations it is quite clear that XRD and PND consider electron distributions from different and complementary perspectives. XRD enables the reconstruction of the total electron distribution, 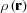, while PND provides information which yields the spin density, 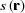. The two quantities can therefore be expressed in terms of spin-resolved electron densities, 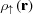 and 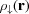, respectively, representing spin up and spin down only electron density distributions
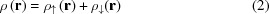


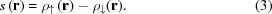



It is therefore obvious that a combined analysis of accurate high-resolution X-ray and polarized neutron diffraction data should yield unprecedented access to spin-resolved electron densities for crystals with significant magnetic properties. Despite such an elementary relationship between spin and charge densities no successful combined analysis has been reported so far. The reason mostly lies in the lack of a common model and the difficulty in finding a fair balance between sets of data obtained from different experiments.

### 4.1. A ‘spin-split’ pseudo-atoms model   

In order to carry out the present work, a ‘spin-split’ pseudo-atoms model was adopted to simultaneously interpret XRD, unpolarized neutron and PND sets of data (Deutsch *et al.*, 2012 ▶). Following the above mentioned H–C model, each atomic contribution is modeled by a frozen core density (identical to that of a similar free atom) and a parameterized valence allowing for contraction/expansion as well as angular distortion with a similar formalism, as explained above. However (and thereby departing from the usual H–C model), when an atomic region is expected to carry a significant spin distribution, the valence part of the model is split into spin up and spin down contributions. The number of parameters in the model is thus moderately increased compared with the usual charge density model for the total density distribution as the magnetization distribution is often limited to a small set of atoms. For an atom expected to carry a magnetic moment, the pseudo-atom contribution 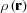 then writes
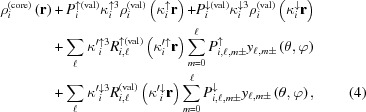
where 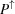 and 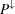 refer to spin up and spin down parameters, respectively. Thus, the challenge is in the determination of 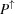 and 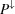, as well as 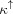 and 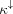, against XRD and PND data in a unique refinement procedure, thanks to the complementarities of the two methods.

Finding a fair way to gather experimental data from different origins still represents a real challenge. No routine strategy has yet been identified and each specific case has to be carefully considered. In our approach it was found that a satisfying result was reached when the following quantity was considered for minimization (Gillet *et al.*, 2001 ▶; Bell *et al.*, 1996 ▶):
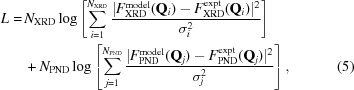
where 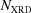 and 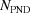 are the number of data points in each set and 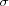 represents the estimated standard deviation for each data point.

A more detailed description of the joint refinement methodology is given in Deutsch *et al.* (2012 ▶).

## 5. First spin-resolved electron density reconstruction and comparison with *ab initio* quantum computations   

### 5.1. A molecular magnet as a test case   

Crystallized molecular magnets are typical examples of large systems with a limited number of magnetic atoms. Thus, the molecular complex Cu_2_ *L*_2_(N_3_)_2_ [*L* = 1,1,1-trifluoro-7-((dimethylamino)-4-methyl-5-aza-3-hepten-2-onato)] recently studied by PND (Aronica *et al.*, 2007 ▶) was chosen as a test case for this joint investigation. This complex belongs to an extensively studied family of dicopper complexes in which the Cu^2+^ ions are coupled by two azido bridges (N_3_^−^). This family is of particular interest in the field of molecular magnetism because the nature of the intramolecular magnetic coupling varies from strongly ferromagnetic to strongly antiferromagnetic coupling depending on the geometry of the bridging ligands. In fact, when two azido groups are symmetrically connected to the Cu atoms by only one N atom (End-On) a triplet ground state is always observed, while a singlet state is observed when these two azide groups relate the Cu atoms by the extremities (End-to-End).

For a long time, the Cu—Cu magnetic interaction mechanism was controversial. It was finally established, partly thanks to PND (Aebersold *et al.*, 1998 ▶), that the ferromagnetic coupling in End-On (EO) complexes is due to the quasi-orthogonality of the magnetic orbitals centred on the Cu atoms for a specified range of values of the Cu—N—Cu bridging angle, while the antiferromagnetic (AF) coupling in End-to-End (EE) complexes originates from the strong overlap between these orbitals.

**Figure 1:**
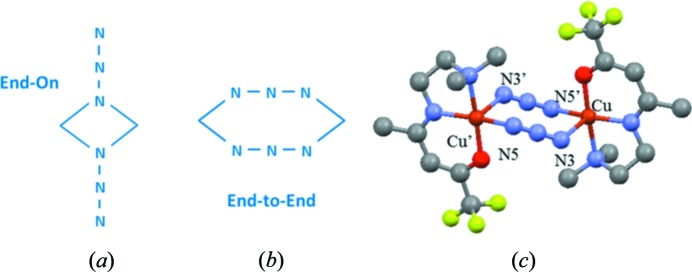
Di-azido copper complexes. Schematic representation of (*a*) End-On and (*b*) End-to-End conformation of di-azido di-Cu complexes. (*c*) View of the Cu_2_ *L*_2_(N_3_)_2_ molecule. N atoms are represented in blue, O in red, C in grey, F in yellow and Cu in orange. H atoms are not shown for reasons of clarity.

In the case of asymmetric bridges, a more subtle interaction mechanism has to be invoked. Indeed, some EO compounds have been shown to exhibit AF coupling and, conversely, ferromagnetic coupling in EE compounds have recently been obtained such as in the present End-to-End Cu_2_ *L*_2_(N_3_)_2_ complex. The crystal structure is formed by discrete neutral centrosymmetric five-coordinated Cu^II^ dinuclear complexes [Cu⋯Cu = 5.068 (1) Å; Aronica *et al.*, 2007 ▶], which are well isolated from each other in the crystal. The azido groups bridge the Cu ions in an asymmetric fashion [Cu—N3 = 2.000 (1) Å, Cu—N5 = 2.346 (1) Å; Fig. 1 ▶].

### 5.2. Results   

Details of the three distinct data collections on single crystals of the molecular complex Cu_2_ *L*_2_(N_3_)_2_ by X-ray, neutron and polarized neutron diffraction, respectively, are reported in Table 1 ▶, as well as the conditions and statistical agreement factors of the joint refinement. For a detailed description of the first XRD and PND data treatment for the copper compound see Deutsch *et al.* (2013 ▶) and Lecomte *et al.* (2011 ▶).

**Figure 2:**
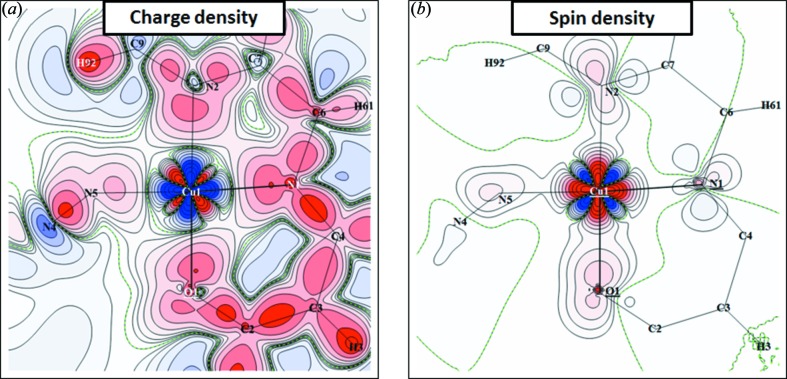
Charge and spin density maps in the plane containing Cu, O1 and N5. (*a*) Static deformation density map obtained by means of the joint refinement strategy. Isocontours are drawn for ± 0.01 × 2^*n*^ e Å^−3^ with *n* = 0–13 (positive red, negative blue). (*b*) Spin density map obtained by means of the joint refinement strategy. Isocontours are drawn for ± 0.01 × 2^*n*^ μB Å^−3^ with *n* = 0–13, spin up contours in red, spin down contours in blue.

The deformation charge density upon the molecule bond formation displayed in Fig. 2 ▶(*a*) was deduced by subtracting the density of independent atoms (computed) from the experimental electron distribution 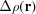 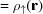 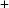 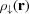 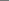 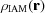 reconstructed from PND and XRD data. Fig. 2 ▶(*b*) shows the experimental spin density 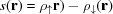 which was obtained taking into account a correction for the orbital contribution to the total magnetization density. This correction was done by applying the usual dipole approximation to describe the orbital form factor (Squires, 1978 ▶) which is written as a sum of the tabulated radial integrals 〈*j*0〉 and 〈*j*2〉 for Cu^2+^ (Brown, 1992 ▶) multiplied by a coefficient 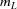 = 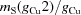, where *g*_Cu_ = 2.175 (Aronica *et al.*, 2007 ▶). A value of 0.07 µ_B_ was taken for this coefficient, according to the refined valence population associated with the orbital form factor in the PND-only refinement.

The charge deformation density map shows the usual distribution of the electrons in all bonds. It describes the *d* electron redistribution around the Cu atom, and the lone-pair densities of N and O atoms of the ligand directed toward the Cu atom. The corresponding spin density exhibits a 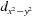-type distribution around the copper with maxima directed toward the N and O atoms and depletion in the bisecting direction. Very little spin density is found in the vicinity of the ligand atoms.

**Figure 3:**
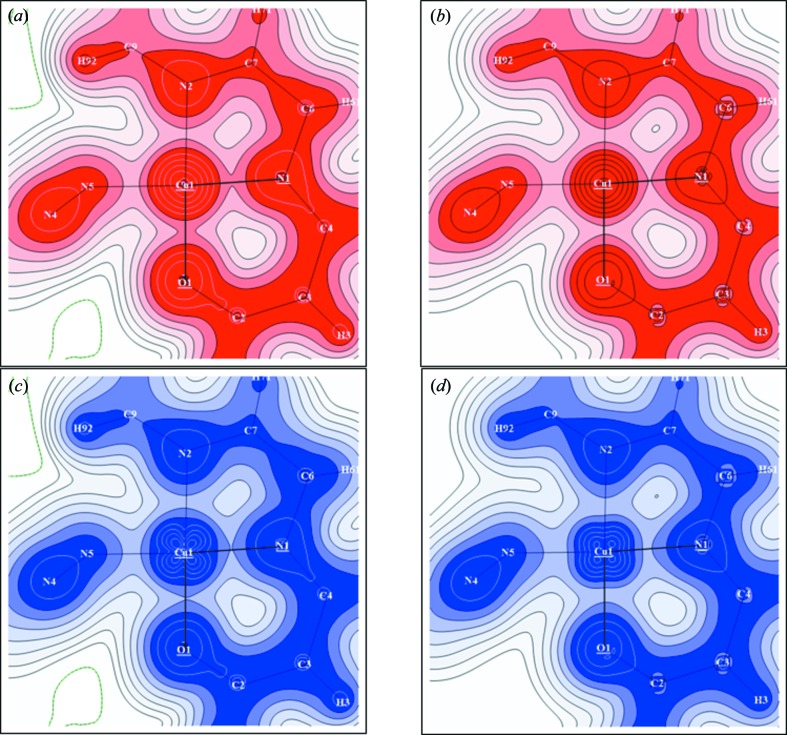
Spin-resolved electron densities. Left: (*a*) Experimental spin up (majority) and (*c*) experimental spin down (minority) valence electron densities from joint refinement of the spin-split model. Right: (*b*) Theoretical spin up (majority) and (*d*) theoretical spin down (minority) valence electron densities from *ab initio* quantum computation. The density distributions are represented in the Cu—N1—O1 plane (contours 0.01 × 2^*n*^ e Å^−3^ (*n* = 0–12)).

**Figure 4:**
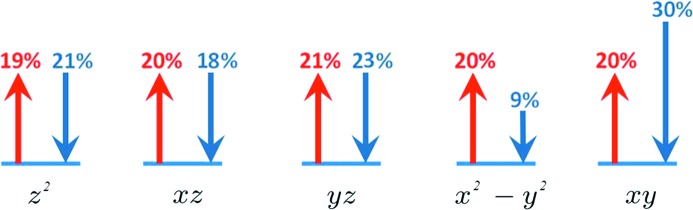
Schematic representation of the Cu *d*-orbital type function populations for up and down electrons. The arrows sizes are proportional to the respective spin populations.

The first spin-resolved valence electron density distributions obtained experimentally from a joint analysis of a common model are displayed in Fig. 3 ▶. As can be clearly observed, the spin-split model obtained from the proposed joint refinement strategy has made it possible to successfully discriminate the density probability distribution of spin up and spin down electrons. Quantum computations (Frisch *et al.*, 2009 ▶), by means of the Density Functional Theory (B3LYP/ 6-31++Gdp), were carried out on an isolated molecule in its experimental geometry. The theoretical and experimental distributions compare extremely well (Fig. 3 ▶); the differences are hardly seen at the drawn contour level. The spin up distribution in the vicinity of the copper nucleus is spherical, while the down spin distribution shows maxima in the bisecting direction of the ligands in both cases (theory and experiments). It thus appears striking that most of the electron anisotropy around the Cu atom (Fig. 3 ▶) should be attributed to spin down electrons. This is indeed confirmed by a *d*-type function analysis (Holladay *et al.*, 1983 ▶) which neglects the covalent part of the metal–ligand bonds of the Cu atom reported in Fig. 4 ▶. It is found that 30% of spin down electrons lie in the 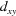-type function with corresponding 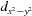 depletion (9%), while all 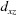, 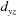 and 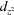 are almost equally populated.

One utmost consequence of the spin-resolved model is that it is shown for the first time that the valence spin ↑ density is 5% more contracted than the spin ↑ density [κ↑ = 0.998 (1), κ↓ = 0.943 (1)]. This is in agreement with theoretical predictions (Watson & Freeman, 1960 ▶) based on a κ refinement of calculated spin-resolved magnetic form factors (Becker & Coppens, 1985 ▶) which indicated a contraction of 1% of the electronic cloud with spin ↑.

**Figure 5:**
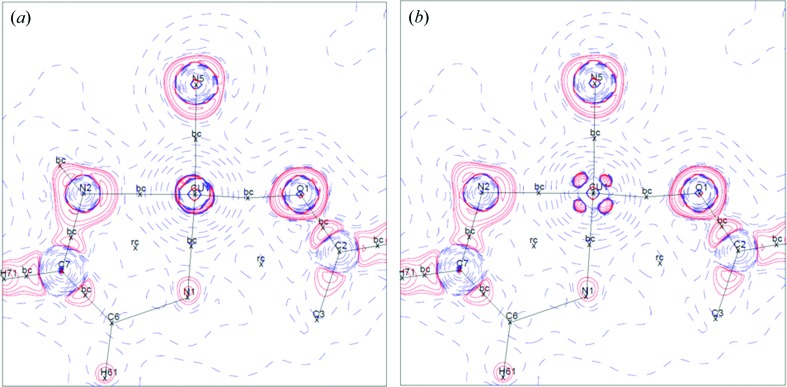
Spin-resolved Laplacian maps in the plane containing Cu, O1 and N5: (*a*) spin up, (*b*) spin down; b.c. for bond critical point and r.c. for ring critical point (saddle point with two positive curvatures).

The topological analysis of the experimental electron density provides a precise description of the interactions in the crystal (Bader, 1990 ▶). It locates particular points, called critical points (CPs), where the gradient of the density vanishes. There are four types of critical points: local maximum of the density, local minimum of the density and two saddle points. The saddle points with two negative curvatures are called bond critical points (BCPs), among them two kinds can be distinguished by the Laplacian value [∇^2^ρ(*r*)]: ‘shared-shell’ interaction, where ρ(CP) is high and ∇^2^ρ(CP) < 0 which means a concentration of electron density at the BCP, and ‘closed-shell’ interaction, where ρ(CP) is low and ∇^2^ρ (CP) > 0 which means a depletion of the electron density at the BCP. The present work using XRD and PND joint refinement allows for the first time the computation of experimental spin-resolved topological properties (Souhassou & Blessing, 1999 ▶). To the best of our knowledge there is only one paper devoted to spin-resolved topological analysis which is a theoretical analysis of b.c.c. and f.c.c. iron (Jones *et al.*, 2008 ▶). The features of the critical points around the Cu atoms are summarized in Table 2 ▶ and the spin-resolved Laplacian maps are displayed in Fig. 5 ▶. The BCPs for the Cu environment are of ‘closed-shell’ type with a small value of the electron density at the BCP. The large positive value of the Laplacian indicates dominant ionic interactions. The positions of the BCPs are almost equal whatever the spin: ρ(CP) and ∇^2^ρ(CP) are slightly higher for spin up electrons, which is both due to a more contracted spin up density and a higher number of spin up electrons (0.74 e more); however, considering the uncertainty on these values (around 10% for the Laplacian) the differences may not be significant.

Moreover, a good agreement between theory and experiment is observed for the net charges as obtained by integrating spin up or down electrons over the corresponding atomic basins for the Cu atom (Table 3 ▶). The majority valence spin state (↑) is experimentally 0.74 e greater than the minority one (↓) compared with the DFT calculation, 0.63 e.

## 6. Conclusion   

It clearly appears that the ‘spin split’ model, together with the proposed joint refinement method, is successful at precisely retrieving all the essential features of the electron distribution with their respective spin dependences. Most important is the dramatic difference between spin up and spin down angular distributions in the vicinity of the copper nuclei. The spin-resolved electron density model confirms for the first time experimentally the different contraction of spin up and spin down radial distribution of Cu atoms as predicted by theory. The topological analysis of the two distributions shows a very good agreement between theory and experiments. We have demonstrated that the joint refinement gives access to the spin up and spin down distributions that were not accessible by standard refinements. This can be applied to any magnetic crystalline material, inorganic or organic. In a wider perspective, this method paves the way for combining different scattering or spectroscopic experiments as electron diffraction, CBED, NQR, NMR, EPR…, that will lead to a more precise and robust description of the electronic behavior in crystalline solids.

## Supplementary Material

Correction for the orbital contribution to the magnetization density. DOI: 10.1107/S2052252514007283/ti5001sup1.pdf

## Figures and Tables

**Table 1:** Details of the data collections and combined refinement (the total number of parameters in the joint refinement is equal to 887, which is smaller than the sum of parameters refined on the different data sets because some parameters are common to different experiments)

	X-ray diffraction	Neutron diffraction	Polarized neutrons
Number of measured reflections	15 731	5049	474
Number of used reflections	7208 (*I* > 3σ, sin θ/λ < 1 Å^−1^)	2303 (*I* > 3σ)	212 (*F*_N_ > 5 × 10^−12^ cm)
Resolution (sin θ/λ)_max_ (Å^−1^)	1.13	0.78	0.5
Temperature (K)	10	30	2
Magnetic field (T)	–	–	5
Wavelength (Å)	0.71073	0.832	0.84
Statistical agreement factors (%)	*R_w_*(*F*) = 1.52	*R_w_*(*F*_N_) = 5.50	*R_w_*(*R*_1_) = 8.96
			*R_w_*(*R*) = 0.98
Number of parameters	626	297	69

**Table 2:** Topological properties of the spin up and spin down densities at critical points in the neighbouring Cu atom

Bond	*d* (Å)	ρ(CP) (e Å^−3^)	∇^2^ ρ(CP) (e Å^−5^)	*d*_1-bcp_ (Å)	λ_1_ (e Å^−5^)	λ_2_ (e Å^−5^)	λ_3_ (e Å^−5^)
Spin up							
Cu—O1	1.936	0.36	6.38	0.966	−2.05	−1.90	10.33
Cu—N1	1.961	0.34	5.90	0.979	−1.65	−1.53	9.09
Cu—N5	2.003	0.31	4.51	1.002	−1.57	−1.52	7.60
Cu—N2	2.036	0.30	4.56	1.004	−1.57	−1.34	7.47
							
Spin down							
Cu—O1	1.936	0.33	5.73	0.965	−1.83	−1.66	9.22
Cu—N1	1.960	0.33	5.48	0.970	−1.59	−1.43	8.50
Cu—N5	2.003	0.29	4.09	0.997	−1.47	−1.39	6.94
Cu—N2	2.037	0.29	4.15	1.000	−1.47	−1.21	6.84

**Table 3:** Experimental copper spin up (*q*↑) and spin down (*q*↓) electron populations (in electrons) integrated over Bader atomic basins

	*q*↑ (e)	*q*↓ (e)	Δ*q* (e)
Experimental	14.55	13.81	0.74
Theoretical	14.29	13.66	0.63

## References

[bb1] Aebersold, M., Gillon, B., Plantevin, O., Pardi, L., Kahn, O., Bergerat, P., von Seggern, I., Tuczek, F., Öhrström, L., Grand, A. & Lelièvre-Berna, E. (1998). *J. Am. Chem. Soc.* **120**, 5238–5245.

[bb2] Aronica, C., Jeanneau, E., El Moll, H., Luneau, D., Gillon, B., Goujon, A., Cousson, A., Carvajal, M. A. & Robert, V. (2007). *Chem. Eur. J.* **13**, 3666–3674. 10.1002/chem.200601253 17285651

[bb3] Bader, R. W. F. (1990). *Atoms in Molecules. A Quantum Theory.* Oxford University Press.

[bb4] Becker, P. & Coppens, P. (1985). *Acta Cryst.* A**41**, 177–182.

[bb5] Bell, B., Burke, J. & Schumitzky, A. (1996). *Comput. Stat. Data Anal.* **22**, 119–135.

[bb6] Brown, P. J. (1992). *International Tables for Crystallography*, edited by A. J. C. Wilson, Vol. C, pp. 512–514. Dordrecht: Kluwer Academic Publishers.

[bb7] Deutsch, M., Claiser, N., Pillet, S., Chumakov, Y., Becker, P., Gillet, J.-M., Gillon, B., Lecomte, C. & Souhassou, M. (2012). *Acta Cryst.* A**68**, 675–686. 10.1107/S0108767312031996 23075610

[bb8] Deutsch, M., Claiser, N., Souhassou, M. & Gillon, B. (2013). *Physics Procedia*, **42**, 10–17.

[bb9] Frisch, M. J. *et al.* (2009). *GAUSSIAN*09, Revision A.1. Gaussian Inc., Wallingford, Connecticut, USA.

[bb10] Gatti, C. & Macchi, P. (2012). Editors. *Modern Charge Density Analysis.* Berlin: Springer.

[bb11] Gillet, J.-M., Becker, P. J. & Cortona, P. (2001). *Phys. Rev. B*, **63**, 235115.

[bb12] Hansen, N. K. & Coppens, P. (1978). *Acta Cryst.* A**34**, 909–921.

[bb13] Holladay, A., Leung, P. & Coppens, P. (1983). *Acta Cryst.* A**39**, 377–387.

[bb14] Jones, T. E., Eberhart, M. E. & Clougherty, D. P. (2008). *Phys. Rev. Lett.* **100**, 017208. 10.1103/PhysRevLett.100.017208 18232817

[bb15] Lecomte, C., Deutsch, M., Souhassou, M., Claiser, N., Pillet, S., Becker, P., Gillet, J.-M., Gillon, B. & Luneau, D. (2011). *Am. Crystallogr. Assoc. Transactions*, http://www.amercrystalassn.Org/documents/2011%20Transactions/Lecomte.pdf

[bb16] Pillet, S., Souhassou, M., Pontillon, Y., Caneschi, A., Gatteschi, D. & Lecomte, C. (2001). *New J. Chem.* **25**, 131–143.

[bb17] Pontillon, Y., Caneschi, A., Gatteschi, D., Grand, A., Ressouche, E., Sessoli, R. & Schweizer, J. (1999). *Chem. Eur. J.* **5**, 3616–3624.

[bb18] Schweizer, J. (2006). *Neutron Scattering from Magnetic Materials*, edited by T. Chatterji, Ch. 4. Amsterdam: Elsevier.

[bb19] Souhassou, M. & Blessing, R. H. (1999). *J. Appl. Cryst.* **32**, 210–217.

[bb20] Squires, G. L. (1978). *Introduction to the Theory of Thermal Neutron Scattering*, p. 139. Cambridge University Press.

[bb21] Watson, R. E. & Freeman, A. J. (1960). *Phys. Rev.* **120**, 1134–1141.

