# Supplementary material for: First spin-resolved electron distributions in crystals from combined polarized neutron and X-ray diffraction experiments
Source: IUCrJ. 2014 Apr 14;1(Pt 3):194–9. doi: 10.1107/S2052252514007283 (PMC4086435; doi:10.1107/S2052252514007283)
Supplement: Supplementary file 1 [file m-01-00194-sup1.pdf]

IUCrJ

Volume 1 (2014)

Supporting information for article:

First spin-resolved electron distributions in crystals from combined polarized neutron and X-ray diffraction experiments

Maxime Deutsch, Béatrice Gillon, Nicolas Claiser, Jean-Michel Gillet, Claude Lecomte and Mohamed Souhassou

The detailed classical treatment of the orbital contribution may be found in [Gillon & Becker, 2012], page 287.

In the independent atom model, the magnetic structure factor of scattering vector **Q** is written as a sum over the atoms i in the cell:

$$F_M\left(\vec{Q}\right) = \sum_{i}^{atoms} m_i f_m^i\left(\vec{Q}\right) e^{i\vec{Q}\vec{R}_i} e^{-W_i}$$

where $f_m^i\left(\vec{Q}\right)$ is the magnetic form factor of atom i which carries a magnetic moment m_i.

In the case of weak spin-orbit coupling, like for a transition metal atom i, the form factor may be written as the sum of a pure spin form factor $f_m^{iS}\left(\vec{Q}\right)$ and a form factor $f_m^{iL}\left(\vec{Q}\right)$ due to the orbital contribution. The magnetic structure factor is then the sum of a pure spin magnetic structure factor $F_M^S\left(\vec{Q}\right)$ and an orbital structure factor $F_M^L\left(\vec{Q}\right)$:

$$F_M\left(\vec{Q}\right) = F_M^S\left(\vec{Q}\right) + F_M^L\left(\vec{Q}\right)$$

The term $F_M^L\left(\vec{Q}\right)$ may be treated as a correction and is taken into account in the theoretical expression of the flipping ratio in the program MOLLYNX.

As described in reference [Squires, 1978] the usual dipole approximation consists in writing the above form factor as:

$$f_m^{iL}\left(\vec{Q}\right) = m_S^i \frac{(g_i - 2)}{g_i}\left(\left\langle j_0^i\right\rangle + \left\langle j_2^i\right\rangle\right)$$

where m^i_S is the magnetic moment of atom i associated with spin, g_i is the Landé splitting factor of atom i and $\left\langle j_\ell^i(Q)\right\rangle$ are the radial integrals for atom i:

$$\left\langle j_\ell^i(Q)\right\rangle = \int_0^\infty r^2 \left(\mathcal{N}_i r_i^{N-1} e^{-\xi_L^i r_i}\right)^2 j_\ell^i(Qr)dr$$

where $\left(\mathcal{N}_i r_i^{N-1} e^{-\xi_L^i r_i}\right)$ is a Slater type radial function with exponent ξ^i_L for the unpaired electron orbital belonging to the atomic shell with quantum numbers N and L and $j_\ell^i(Qr)$ are the spherical Bessel functions of order ℓ = 0, 2 which are tabulated for the 3d elements in [Brown, 1992].

The orbital magnetic structure factors $F_M^L\left(\vec{Q}\right)$ were calculated using the magnetic form factor $\left(\langle j_0\rangle + \langle j_2\rangle\right)$ for Cu^{2+} and a coefficient:

$$m_S^{Cu}\frac{(g_{Cu} - 2)}{g_{Cu}} = 0.07\mu_B$$

with m_S^{Cu} = 0.7μ_B and g_{Cu} = 2.175 [Aronica, 2007]. This estimation was confirmed by refinement of the valence population associated with the orbital form factor introduced in the PND-only refinement.

- B. Gillon & P. Becker, Chapter 8, «Magnetization densities in material science» in *Modern charge density analysis*, Eds C. Gatti and P. Macchi, Springer, (**2012**).
- Squires G. L. in *Introduction to the theory of thermal neutron scattering* (Cambridge: University Press, **1978**), p. 139.
- Brown, P. J., Magnetic form factors, chapter 4.4.5, International tables for crystallography (**1992**) vol. C (A. J. C. Wilson, ed.), pp. 391-399.
- Aronica, C., Jeanneau, E., El Moll, H., Luneau, D., Gillon, B., Goujon, A., Cousson, A., Carvajal, M. A. & Robert, V. (2007). *Chem. Eur. J.* **13**(13), 3666–3674.
